# Global DNA Hypomethylation in Peripheral Blood Leukocytes as a Biomarker for Cancer Risk: A Meta-Analysis

**DOI:** 10.1371/journal.pone.0034615

**Published:** 2012-04-11

**Authors:** Hae Dong Woo, Jeongseon Kim

**Affiliations:** Cancer Epidemiology Branch, National Cancer Center, Goyang-si, Korea; Dartmouth College, United States of America

## Abstract

**Background:**

Good biomarkers for early detection of cancer lead to better prognosis. However, harvesting tumor tissue is invasive and cannot be routinely performed. Global DNA methylation of peripheral blood leukocyte DNA was evaluated as a biomarker for cancer risk.

**Methods:**

We performed a meta-analysis to estimate overall cancer risk according to global DNA hypomethylation levels among studies with various cancer types and analytical methods used to measure DNA methylation. Studies were systemically searched via PubMed with no language limitation up to July 2011. Summary estimates were calculated using a fixed effects model.

**Results:**

The subgroup analyses by experimental methods to determine DNA methylation level were performed due to heterogeneity within the selected studies (p<0.001, I^2^: 80%). Heterogeneity was not found in the subgroup of %5-mC (p = 0.393, I^2^: 0%) and LINE-1 used same target sequence (p = 0.097, I^2^: 49%), whereas considerable variance remained in LINE-1 (p<0.001, I^2^: 80%) and bladder cancer studies (p = 0.016, I^2^: 76%). These results suggest that experimental methods used to quantify global DNA methylation levels are important factors in the association study between hypomethylation levels and cancer risk. Overall, cancer risks of the group with the lowest DNA methylation levels were significantly higher compared to the group with the highest methylation levels [OR (95% CI): 1.48 (1.28–1.70)].

**Conclusions:**

Global DNA hypomethylation in peripheral blood leukocytes may be a suitable biomarker for cancer risk. However, the association between global DNA methylation and cancer risk may be different based on experimental methods, and region of DNA targeted for measuring global hypomethylation levels as well as the cancer type. Therefore, it is important to select a precise and accurate surrogate marker for global DNA methylation levels in the association studies between global DNA methylation levels in peripheral leukocyte and cancer risk.

## Introduction

Epigenetic processes are important in development and cell differentiation, and can be altered by environment, diet, and aging [1], [2]. DNA methylation, which is a major epigenetic mechanism, is involved in various biological processes including cancer [3]–[6]. DNA hypomethylation is an early event in human carcinogenesis [7]–[9] and is associated with genetic instability in cancer cells [10], [11]. Methylation levels of DNA are maintained by DNA methyltransferases (DNMTs). Dnmt3a and Dnmt3b are responsible for de novo DNA methylation [12]–[14]. Thus inactivation of DNMTs causes global hypomethylation of the genome or hypomethylation of specific families of repeated sequences [14]–[16]. However, mechanisms of DNA hypomethylation are not fully understood. There are several mechanisms accounting for DNA demethylation. Direct removal of the methyl group by methyl CpG binding domain protein 2 (MBD2) has been reported [17], although this result has not been confirmed by other authors [18], [19]. GADD45A (growth arrest and DNA-damage-inducible, alpha) is associated with repair-mediated DNA demethylation [20]. However, the work of Jin et al. [21] does not confirm this association. DNA repair enzymes may demethylate DNA [22], and direct evidence of base excision repair (BER) mediated DNA demethylation has been proposed [23]. Brother of the regulator of imprinted sites (BORIS) expression is associated with demethylation [24]. Woloszynska-Read et al. [25] has suggested that the ratio of BORIS/CCCTC-binding factor (CTCF) expression is related to DNA demethylation. Because direct removal of the methyl group from the 5′ position of the cytosine is unfavorable, studies suggesting natural mechanisms of DNA demethylation have been inconsistent.

The CG sequences of the promoter region are normally unmethylated to allow gene expression, whereas mammalian DNA repeats are highly methylated in somatic tissues [12], [26]. DNA hypermethylation in tumor suppressor gene (TSG) promoters causes repression of TSGs. Hypomethylation of unique or repeated DNA sequences may affect chromatin structure and genomic instability, lead to transcription activation, and increase expression of cancer-promoting genes [26]. Both DNA hypomethylation and hypermethylation have been observed in human cancer, but hypomethylation of DNA, especially in repetitive elements, are more frequently associated with cancer, resulting in net losses of 5-methylcytosine (5-mC) [26].

The association between global hypomethylation of tumor DNA and cancer risk has been demonstrated in various human tumors [27]–[30]. In addition, global hypomethylation of DNA in various cancer and adjacent normal tissues has been detected [31], [32]. Therefore, many investigators have studied DNA methylation as a biomarker for cancer screening. Early detection of cancer results in better prognosis. However, harvesting tumor tissue is invasive and cannot be routinely performed. Therefore, the association between cancer risk and global DNA hypomethylation levels in blood leukocytes has been investigated. We performed a meta-analysis to estimate overall cancer risk according to global DNA hypomethylation levels among studies with various cancer types and analytical methods used to measure DNA methylation.

## Methods

### Study selection

We systemically searched for studies via the electronic databases using PubMed with the terms “cancer risk and (methylation or hypomethylation) and (blood or leukocyte)” with no language limitation up to July 2011. A manual search with a reference list of selected journals was performed. However, no new articles meeting the inclusion criteria were identified. The inclusion/exclusion criteria were as follows: (1) the original article with case-control or cohort designs; (2) peripheral blood leukocytes were used to measure global DNA methylation levels; (3) patients who were newly diagnosed with cancer in case-control studies, and blood was collected in participants who were free of cancers at baseline in cohort studies; (4) the studies with gene-specific DNA methylation were excluded; and (5) the study reported 95% confidence intervals (CI) with adjusted odds ratios (OR) or relative risks (RR) for cancer risk in subjects with the lowest level of global DNA methylation compared to that in patients with the highest level of global DNA methylation. If the reference cell contained the lowest level of global DNA methylation, inversed OR and 95% CI was used.

### Data collection

Searched studies were independently reviewed by two investigators (H.D.W and J.K.). Studies with eligible data for meta-analysis contained information on authors, publication year, experimental methods to measure global DNA methylation levels, cancer sites and types, country where the study was performed, study period, number of cases and controls, categories of global DNA methylation levels, adjusted OR/RR and 95% CI, p-values for trends, and confounding variables were considered. Adjusted OR/RR and 95% CI were selected to exclude confounding effects and to include the studies that did not report the number of cases and controls for each category.

### Statistical analysis

All statistical analyses were performed using the STATA software package (version 10, College Station, TX). Log point effect estimates and the corresponding standard errors were calculated using covariate-adjusted point effect estimates and 95% CI from selected studies and weighted by the inverse variance to calculate summary estimates [33]. The heterogeneity test across studies was measured using the Q-test based on the χ^2^ statistic, considering significant statistical heterogeneity as p<0.05, and the I^2^ test according to Higgins et al. [34]. Subgroup analyses were conducted by experimental methods to measure global DNA methylation levels or based on cancer type due to between-study heterogeneity. A fixed effect model was used in the meta-analysis because a random effect model is less conservative than a fixed effect model by giving more weight to small sample studies, which are more likely to have publication bias, especially when small numbers of the studies were combined [35], [36].

## Results

**Figure 1:**
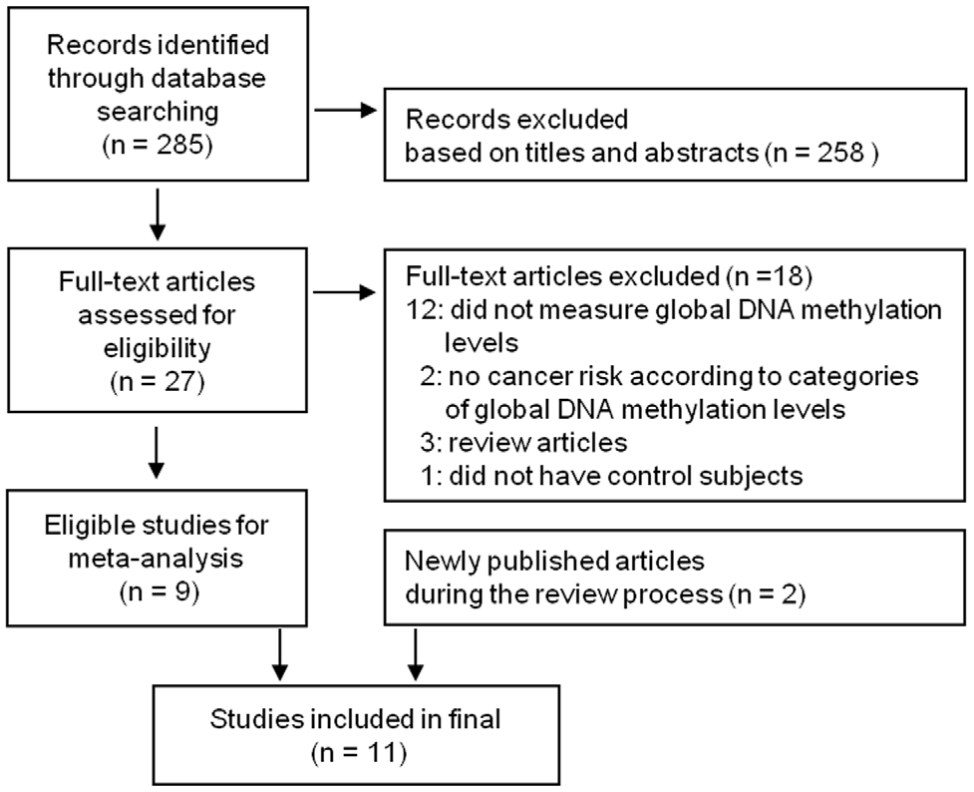
Flow diagram of study selection.

A total of 258 studies were excluded in the first pass based on titles and abstracts among 285 searched articles. The remaining 27 studies were further reviewed. Eighteen studies that did not meet inclusion criteria were excluded for the following reasons: 12 studies did not measure global DNA methylation levels; two studies did not report the cancer risk according to categories of global DNA methylation levels; 3 studies were review articles; and 1 study did not have control subjects. Eleven studies comprising ten case-control studies and 1 cohort study were finally selected (Table 1) [37]–[47]. We found 2 studies that were newly published during the review process [45], [46]. Because only a limited number of studies met the criteria for our meta-analysis, we decided to include these 2 additional studies (Figure 1). Because Pufulete et al. [37] have reported both the risks of colorectal cancer and colorectal adenoma, twelve cases were used for meta-analysis. Three bladder cancer, two colorectal adenomas, one colorectal cancer, one breast cancer, two gastric cancer, one head and neck squamous cell carcinoma (HNSCC), one renal cell cancer, and one overall cancer case were included. Zhu et al. [47] have reported the risks of all cancer as well as each type of cancers categorized into two and four group of global DNA hypomethylation levels. However, the data from all cancer risk with two categories were selected due to the small number of cancer incidence. Lim et al. [39] and Liao et al. [45] have reported OR and 95% CI in people of the highest tertile of genomic methylation compared to those in the lowest tertile of genomic methylation. Therefore, we used the inversed OR and 95% CI to calculate the pooled estimate. Egger's test for publication bias showed non-significant results (p = 0.483).

**Table 1:** Studies on global DNA hypomethylation in peripheral blood leukocytes and cancer risk included in the meta-analysis.

Author (Year)	Methods	Cancer site	Country	Study period	No. cases/controls	Category	OR (95% CI)	P trend	Confounding variables that were considered
Case-control studies									
Pufulete	Methyl	Colorectal	UK	2000	76/	T1 (Adenoma)	1	0.01	Age, sex, body mass index,
(2003)^(36)^	acceptance			−2001	(35 adenoma	T2	6.68 (0.99–45.12)		smoking status, and alcohol
	assay				and 28 cancer)	T3	10.27 (2.05–51.46)		intake
						T1 (Cancer)	1	0.08	
						T2	2.98 (0.51–17.40)		
						T3	4.90 (0.84–28.53)		
Hsiung	LINE-1	HNSCC	USA	1999	278/526	T3 (>76.9)	1	0.03	Age, sex, race, smoking
(2007) ^(37)^	(LRE1)			−2003		T2 (73.6-0.76.9)	1.3 (0.9–2.0)		history, lifetime average
						T1 (<73.6)	1.6 (1.1–2.4)		alcoholic drinks weekly,
									and positive HPV16 serology
LIM	%5-mC	Colorectal	USA	2000	115/115	T3 (5.43–6.42)	1†	0.002	Matching factors (age and
(2008) ^(38)^		Adenoma		−2002		T2 (5.29–5.43)	0.39 (0.66–2.94)		month of blood draw),
						T1 (2.76–5.29)	5.88 (2.04–16.67)		smoking history, and red
									meat intake
Moore	%5-mC	Bladder	Spain	1998	775/397	Q4 (≥3.68)	1	<0.001	Age, sex, region, smoking
(2008) ^(39)^				−2001		Q3 (3.19–3.68)	2.05 (1.37–3.06)		status, pack-years, interaction
						Q2 (2.46–3.19)	1.62 (1.07–2.44)		(smoking status, pack-year)
						Q1 (<2.46)	2.67 (1.77–4.03)		
Choi	%5-mC	Breast	USA		179/180	T3	1	<0.001	Age, race, and batches
(2009) ^(40)^						T2	1.49 (0.84–2.65)		
						T1	2.86 (1.65–4.94)		
Hou	LINE-1,	Gastric	Poland	1994	302/421	T3 (81.7–90.4)	1	0.12	Age, sex, educational level,
(2010) ^(41)^	Alu			−1996		T2 (78.6–81.7)	1.2 (0.8–1.8)		smoking, and alcohol
						T1 (67.6–78.6)	1.4 (0.9–2.0)		
Wilhelm	LINE-1	Bladder	USA	1994	285/465	74.25–91.96 (90%)	1		Age and sex
(2010) ^(42)^				−1998		57.89-74.25 (10%)	1.8 (1.12–2.90)		
Cash	LINE-1	Bladder	China	1995	510/528	T3 (≥82.52)	1	0.268	Age at reference date, sex,
(2011) ^(43)^				−1998		T2 (81.22–82.52)	1.10 (0.81–1.50)		and family history of cancer
						T1 (<81.22)	1.28 (0.95–1.73)		
Liao^(44)^	LINE-1	Renal cell	Central	1999	328/654	Q4 (86.0–90.2)	1†	0.004	Age, sex, center, smoking status
(2011)			and	−2003		Q3 (81.7–83.0)	0.58 (0.38–0.90)		BMI, high blood pressure, and
			Eastern			Q2 (80.3–81.7)	0.54 (0.36–0.83)		vegetable intake
			Europe			Q1 (78.4–83.6)	0.49 (0.32–0.75)		
Nested case-control study									
Gao^(45)^	LINE-1,	Stomach	China	1997	192/384	Q4	1		Age
(2011)	Alu			−2009		Q3	0.73 (0.44–1.21)		
						Q2	1.02 (0.64–1.60)		
						Q1	0.89 (0.54–1.46)		
Cohort studies				Incidence/total					
Zhu	LINE-1,	All	USA	1963	29/517	High (86.2-77.2)	1		Age, BMI, race, education,
(2011) ^(46)^	Alu			−1999		Low (77.1-68.1)	3.0 (1.3–6.9)		smoking status, pack-years,
									and alcohol drinking

† OR (95% CI) was recalculated because the reference was the lowest tertile of genomic methylation in the original result.

T: tertile, Q: quartile, %5-mC: percentage of 5-methyl cytosine, LINE-1: long interspersed nucleotide element 1, LRE1: LINE retrotransposable element 1.

**Figure 2:**
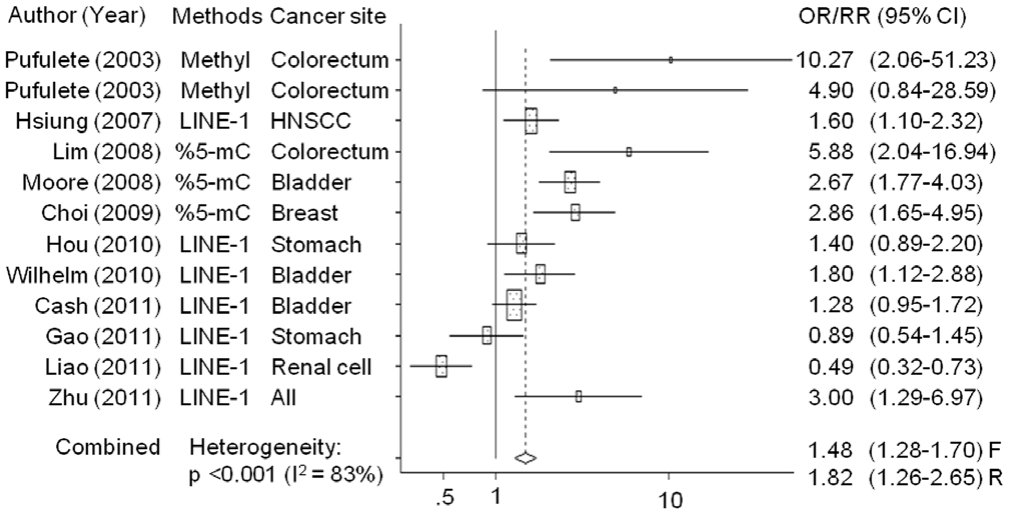
Forest plot of the association between cancer risks and global DNA hypomethylation in peripheral blood leukocytes. Colorectal A: Colorectal Adenoma, Methyl: Methyl acceptance assay, LINE-1: Long interspersed nuclear elements, and %5-mC: Percentages of 5-methylcytosine. F: Fixed effects model, R: random effects model. The horizontal lines through the boxes represent 95% confidence intervals (CI). The centers of the boxes are situated in the point estimate (OR/RR), and the bigger boxes mean studies have relatively greater influence in the summary estimates.

**Figure 3:**
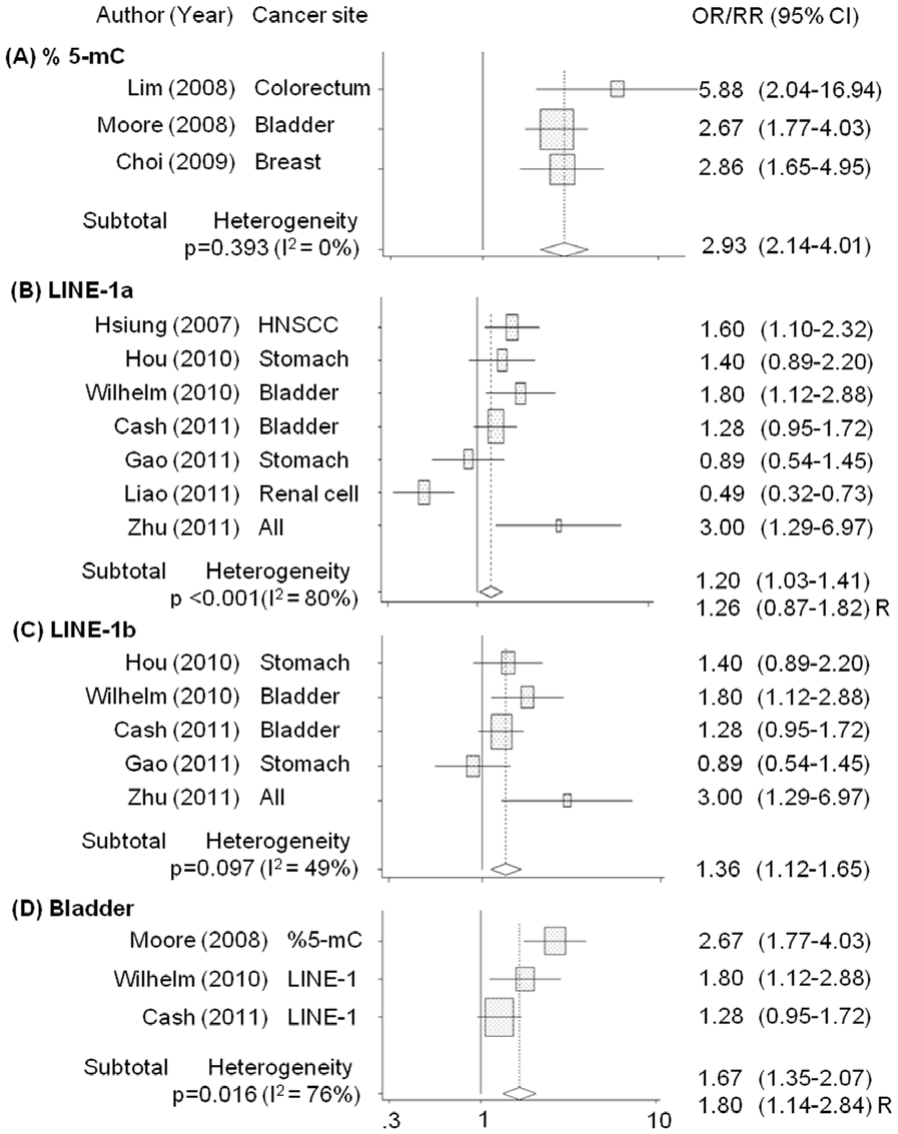
Subgroup analysis of the association between cancer risks and global DNA hypomethylation in peripheral blood leukocytes. (A) %5-mC, (B) LINE-1, and (C) LINE-1 used same target sequence. The association between bladder cancer risk and global DNA hypomethylation (D). R: random effects model. Summary estimates were calculated based on a fixed effects model, unless otherwise stated.

Figure 2 shows the meta-analysis of the selected studies. Global DNA hypomethylation was associated with increased cancer risk (OR: 1.48, 95% CI: 1.28–1.70, p<0.001). However, between-study heterogeneity was significantly high (p<0.001, I^2^: 83%). Therefore, we conducted meta-regression using experimental methods used to determine global DNA methylation levels [methyl acceptance capacity of DNA, percentage of 5-methylcytosine (%5-mC) vs. long interspersed nucleotide element 1 (LINE-1)], cancer sites (colorectal, stomach, others vs. bladder), and sex (male, total vs. female). Experimental methods were significantly different from each other (%5-mC vs. LINE-1: p = 0.011) when sex and experimental methods were included in the meta-regression model. However, no significant differences were observed when cancer site was further included. Four population-based studies were identified, including 1 nested case-control study; all the studies showed homogeneous summary estimates (OR [95% CI] = 1.36 [1.12–1.64]; heterogeneity test: p = 0.159, I^2^ = 42%). However, the experimental methods of these 4 studies, which involved LINE-1 analysis and hospital-based investigations, still showed significant heterogeneity. The meta-regression data did not show any differences with regard to inclusion of the study design in the analysis. Therefore, we did not include the study design in our analysis. Subgroup analyses were performed by experimental methods (%5-mC, LINE-1) and studies of bladder cancer to further explore the variance among studies (Figure 3). Heterogeneity among studies was not detected on %5-mC method (p = 0.393, I^2^ = 0%), but considerable variance was found in the subgroup analysis of LINE-1 method (p<0.001, I^2^: 80%) and bladder cancer (p = 0.016, I^2^: 76%). The group with the lowest global DNA methylation levels had significantly higher cancer risks compared to the group with the highest global DNA methylation levels in %5-mC and LINE-1 studies [OR (95% CI): 2.93 (2.14–4.01) and 1.20 (1.03–1.41), respectively]. In the LINE-1 subgroup, with the exception of the study by Hsiung et al. [38] and Liao et al. [45] which used different region of DNA target sequence from other studies, I^2^ statistics were 49% (p = 0.097) and global DNA hypomethylation levels were significantly associated with increased cancer risk [OR (95% CI): 1.36 (1.12–1.65)].

## Discussion

Eleven studies were used in the present study to estimate overall results. These studies have tested whether global DNA methylation level in peripheral blood DNA is a good marker to detect cancer. Global DNA hypomethylation of blood leukocytes was associated with increased cancer risk. However, the association varied according to the experimental methods used and region of DNA targeted for measuring global hypomethylation levels as well as the cancer type.

Three experimental methods were identified to measure global DNA methylation levels in the present meta-analysis: Three studies used %5-mC; seven studies used LINE-1 with pyrosequencing (6 studies) and a modified version of the combined bisulfite restriction analysis of the LINE retrotransposable element 1(LRE1) sequence (1 study); one study analyzed the methyl acceptance capacity of DNA using [3H-methyl] S-adenosylmethionine. Subgroup analysis in % 5-mC method was homogeneous. However, LINE-1 method and bladder cancer, which was the only cancer type that was analyzed in more than 3 studies, remained significantly heterogeneous. Many analytical methods have been developed to determine DNA methylation levels [48]–[51]. Methyl acceptance assay is based on the capacity of radio-labeled methyl incorporation into DNA, thus high methyl group incorporation indicates lower levels of DNA methylation. However, high day-to-day variation and inaccurate DNA concentration due to the difficulty of mixing genomic DNA solution homogenously have been observed [52]. The direct measurement of percentages of 5-methylcytosine was used in many epidemiological studies to estimate global DNA methylation contents using reversed-phase high-performance liquid chromatography (HPLC), liquid chromatography (LC)-mass spectrometry, or high-performance capillary electrophoresis (HPCE). These methods are highly quantitative and reproducible, but they are not suitable for epidemiological studies with large sample size and high amount of DNA are required to yield reliable results. Introduction of sodium bisulfite conversion of genomic DNA has revolutionized the analytical methods in methylation analysis [13]. In addition, pyrosequencing which is a high-throughput and accurate method is currently available [51]. Repetitive sequences comprise large portions of the human genome and are CpG-rich [53], [54]. Repetitive genomic regions such as LINE-1 and Alu are usually methylated in somatic tissues [55]. Therefore, pyrosequencing with bisulfite-treated DNA to measure repetitive element methylation has been used as surrogate markers for global DNA hypomethylation.

However, in the study of Choi et al. [41], global DNA methylation levels were measured with %5-mC and LINE-1 in a pilot study. However, both methods did not correlate with each other, and the LINE-1 methylation level showed no significant differences between cases and controls. No statistical significant correlation between %5-mC and LINE-1 might be due to the low sample size in a pilot study, but correlation coefficient was still very small (r = −0.204). Therefore, LINE-1 may not be appropriate to serve as a sensitive surrogate marker for global DNA methylation. In addition, Nelson et al. [56] reported that methylation levels with LINE-1 method can be varied depending on the target CpG sequence and across samples. The CpG sequence frequently used for LINE-1 is located in the 5′ region which is often deleted without knowing the exact frequency; consequently the numbers of elements used for LINE-1 measurement can be different across samples. Phokaew et al. [57] reported that LINE-1 methylation levels of white blood cells and normal oral epithelium were highly variable depending on where the targeted sequence are located, but similar pattern was observed in the same locus. This result suggests that targeting locus for LINE-1 methylation measurement in normal tissues should be cautiously selected, but the amount of variation of methylation levels across samples may not be large. Five out of 7 studies of involving LINE-1 methylation in our study used the same target sequence for LINE-1, and they were all referenced from Bollati et al. [58]. The summery estimates of these studies showed little heterogeneity and showed significant association with cancer risk. Liao et al. [45] reported global DNA methylation levels at 4 different positions; however, associations with cancer risk were different in each position. Because the studies were performed at different cancer sites, it cannot be concluded whether experimental methods were more important than the cancer type in the association between cancer risk and global DNA methylation using peripheral blood. However, these results suggest that the experimental methods used to quantify global DNA methylation levels are important factors in the association study with cancer risk.

The effects of aberrant DNA methylation of promoter regions on cancer risk are relatively clear. However, the relationship between global DNA hypomethylation and cancer are not conclusive. Global DNA hypomethylation is related to early stages of carcinogenesis [7]–[9], tumor progression [29], or both [59]. Global hypomethylation may function as a cause or consequence of carcinogenesis. However, the present results could not confirm the association. Most studies (10 out of 11 studies) had a case-control design. The only cohort study in the meta-analysis had small cancer incidence cases and showed inconsistent results with different methods.

DNA hypomethylation in blood leukocytes of patients with cancer might be caused by circulating tumor DNA [60]. However, the effects of tumor DNA on the results of the present study seem to be minimal. Active and aggressive tumors release tumor DNA but hypomethylation can be found in early stages of cancer, and tumor DNA is detected in the plasma of cancer patients but the methylation levels were assessed using blood leukocyte.

The limitation of this study is that a small number of publications met the criteria for the present meta-analysis and that these articles were mostly case-control designed studies. Although global DNA hypomethylation in blood leukocytes was associated with increased cancer risk in the meta-analysis, global DNA hypomethylation may be useful as a biomarker for cancer susceptibility but not a diagnostic tool for cancer. Because it is difficult to decide the cut-off point of hypomethylation for a biomarker for cancer risk, the sensitivity and specificity of global hypomethylation regarding cancer risks could not be determined.

In summary, global DNA hypomethylation in peripheral blood leukocytes may be considered as a biomarker for cancer risk. However, the association with cancer risk may vary with experimental methods, and targeted region of DNA to measure global hypomethylation levels, and cancer type. Numerous methods are available to measure global DNA methylation but are limited for epidemiological studies, which require techniques that are high-throughput, accurate, and economical. Further investigation is needed to elucidate surrogate markers for global DNA methylation levels and to determine whether global DNA hypomethylation is a marker of cancer risk with large cohort studies.
